# Independent and Combined Effects of Lactitol, Polydextrose, and *Bacteroides thetaiotaomicron* on Postprandial Metabolism and Body Weight in Rats Fed a High-Fat Diet

**DOI:** 10.3389/fnut.2016.00015

**Published:** 2016-06-08

**Authors:** Kaisa Olli, Markku T. Saarinen, Sofia D. Forssten, Mari Madetoja, Karl-Heinz Herzig, Kirsti Tiihonen

**Affiliations:** ^1^DuPont Nutrition and Health, Global Health & Nutrition Science, Kantvik, Finland; ^2^Made Consulting, Turku, Finland; ^3^Medical Research Center Oulu, Institute of Biomedicine and Biocenter of Oulu, Oulu University Hospital, University of Oulu, Oulu, Finland; ^4^Department of Gastroenterology and Metabolism, Poznan University of Medical Sciences, Poznan, Poland

**Keywords:** *Bacteroides*, insulin, lactitol, obesity, polydextrose, PYY, satiety signals, triglycerides

## Abstract

Obesity is related to the consumption of energy-dense foods in addition to changes in the microbiome where a higher abundance of gut Bacteroidetes can be found in lean subjects or after weight loss. Lactitol, a sweet-tasting sugar alcohol, is a common sugar-replacement in foods. Polydextrose (PDX), a highly branched glucose polymer, is known to reduce energy intake. Here, we test if the combined effects of lactitol or PDX in combination with *Bacteroides* species will have a beneficial metabolic response in rats fed a high-fat (HF) diet. A total of 175 male Wistar rats were fed either a LF or HF diet. *Bacteroides thetaiotaomicron* (10^10^ bacteria/animal/day) was orally administered with or without lactitol (1.6−2 g/animal/day) or PDX (2 g/animal/day) for 8 days. Postprandial blood samples, cecal digesta, and feces were collected on the last day. Measurements included: body weight, feed consumption, cecal short-chain fatty acids, fecal dry matter and heat value, blood glucose, insulin, triglyceride, and satiety hormone concentrations. Lactitol and PDX decreased the mean body weight when administered with *B. thetaiotaomicron* or when lactitol was administered alone. Levels of postprandial plasma triglycerides declined with lactitol and PDX when administered with *B. thetaiotaomicron*. For intestinal hormone release, lactitol – alone or with *B. thetaiotaomicron* – increased the release of gastrointestinal peptide tyrosine tyrosine (PYY) as well as the area under the curve (AUC) measured for PYY (0–8 h). In addition, levels of insulin AUC (0–8 h) decreased in the lactitol and PDX-supplemented groups. Lactitol and PDX may both provide additional means to regulate postprandial metabolism and weight management, whereas the addition of *B. thetaiotaomicron* in the tested doses had only minor effects on the measured parameters.

## Introduction

Managing postprandial glucose and lipid responses can decrease the risk of metabolic diseases and influence body weight management. Therefore, low glycemic and satiety-increasing food components, such as non-starch polysaccharides, have been studied for their impact on energy metabolism and satiety (1). Several studies have also demonstrated that the gut microbiota contributes to the control of body weight and energy homeostasis (2, 3).

Lactitol, a sweet-tasting sugar alcohol that consists of galactose and sorbitol, is commonly used in low-calorie products to replace sucrose. Lactitol is not absorbed in the small intestine or hydrolyzed by gastrointestinal tract enzymes but, unlike polydextrose (PDX), it is metabolized rapidly by the gastrointestinal microbes [for review, see Ref. (4)]. PDX is a highly branched, randomly bonded glucose polymer and its structural complexity prevents its hydrolysis by mammalian enzymes in the upper gastrointestinal tract. PDX is widely recognized as a soluble fiber (5) and due to its very low energy density, PDX might reduce energy intake and influence the gastrointestinal microbiome. As PDX passes through the small intestine, it is partially metabolized by the colonic microbes (6). A sustained degradation of PDX has been demonstrated also throughout a four-stage colon simulation model (7). In human studies, PDX has already shown to enhance satiety and reduce energy intake during a sequential *ad libitum* lunch (8–10).

Most of the ingested lactitol and part of the PDX are fermented by the microbiota in the lower gastrointestinal tract producing short-chain fatty acids (SCFAs) (4, 7, 11), which stimulate gastrointestinal peptide secretion, such as peptide tyrosine tyrosine (PYY), glucagon-like peptide-1 (GLP-1), and cholecystokinin (CCK) from enteroendocrine cells (12, 13). These peptides control intestinal secretion and motility, i.e., function as ileal brake, in addition to the central inhibition of food intake. PYY has also shown to increase energy expenditure and fat oxidation in humans and rodents (14). Previous animal studies have shown that lactitol stimulates PYY and GLP-1 secretion (15). Lactitol and synbiotics, such as *Lactobacillus delbrueckii* subsp. *rhamnosus* strain GG, and *Bifidobacterium lactis* Bp12 have increased plasma PYY concentrations in animals (15, 16). SCFAs injected directly into the rat colon have been shown to increase the release of PYY (17, 18). In humans, the oral intake of SCFAs and colonic delivery of SCFAs via fermentable dietary fiber stimulated PYY and GLP-1 release, although contradictory effects on appetite and satiety sensation have been observed (19, 20).

It is now well established that obesity is associated with changes in the microbiome, especially the relative abundance of the two dominant bacterial divisions, the Firmicutes and the Bacteroidetes. The gut microbiota of genetically obese (ob/ob) mice had an increased abundance of Firmicutes over Bacteroidetes (21). An increase in the relative abundance of Bacteroidetes has been correlated to weight loss (22). Evidently, the high-fat (HF)-enriched Western diet provides a competitive advantage to the Firmicutes (23). Cani et al. have proposed that the amount of beneficial bifidobacteria in the gut differ according to the state of obesity, showing lower bacterial counts in obese and higher counts in lean subjects (24). Both lactitol and PDX possess prebiotic properties, and lactitol can increase the quantity of beneficial bacteria, such as lactobacilli and bifidobacteria (25). An increase in the number of these beneficial intestinal bacteria has also been demonstrated in human trials after the use of PDX alone (4 g/day) (26) and in combination with a probiotic bacteria mixture (27) or another prebiotic (28). The effect of Bacteroidetes on satiety is still unclear, although the higher relative abundance of gut Bacteroidetes is repeatedly being reported with lean subjects or after weight loss (29). In a recent study in rats, the addition of prebiotics was able to normalize the reduced amount of Bacteroidetes often reported in obesity (30). Prebiotics have also raised the concentration of plasma PYY and the cecum mRNA levels of PYY (31).

Therefore, we tested the combined effects of two carbohydrates and *Bacteroides* species in a large animal trial hypothesizing that the supplementation of *Bacteroides* inoculum might affect the weight gain in rats fed a HF diet, Furthermore, this effect could be modified by the addition of synbiotic effects of lactitol or PDX. The use of a combination of intestinal *Bacteroides* species, *Bacteroides thetaiotaomicron*, and two different indigestible carbohydrates, such as lactitol or PDX, has not been studied before.

## Materials and Methods

### Animals and Experimental Design

A total of 175 male Wistar (HsdBrlHan:WIST) rats (Harlan, The Netherlands) were used in this study. The experiments were approved by the Regional State Administrative Agency of Southern Finland and were conducted under the animal license number ESLH-2008-03964. Institutional and national guidelines for the care and use of animals were followed. Animals arrived at the age of approximately 8 weeks were housed in plastic cages (Makrolon 3, Tecniplast, Bayer MaterialScience AG, Leverkusen, Germany), two to three per cage (floor area 814 cm^2^), and were kept in a conventional animal room with reversed artificial 12 h light/dark cycle (21 ± 3°C, 55 ± 15% humidity). The animals were randomly assigned to different groups and they were acclimatized with their respective diets for 2 weeks before the first dosage of test items. The animals were trained to consume their food within 5 h from the start of the dark cycle.

The first experiment (Experiment A) studied whether *Bacteroides* supplementation can affect satiety and how the responses differ between animals fed a low-fat (LF) or a HF diet. The latter experiment (Experiment B) evaluated the effects of lactitol and PDX – administered separately, or together with *B. thetaiotaomicron* inoculum – on weight gain, satiety signals, and other metabolic parameters in rats fed a HF diet.

#### Experiment A

**Figure 1:**
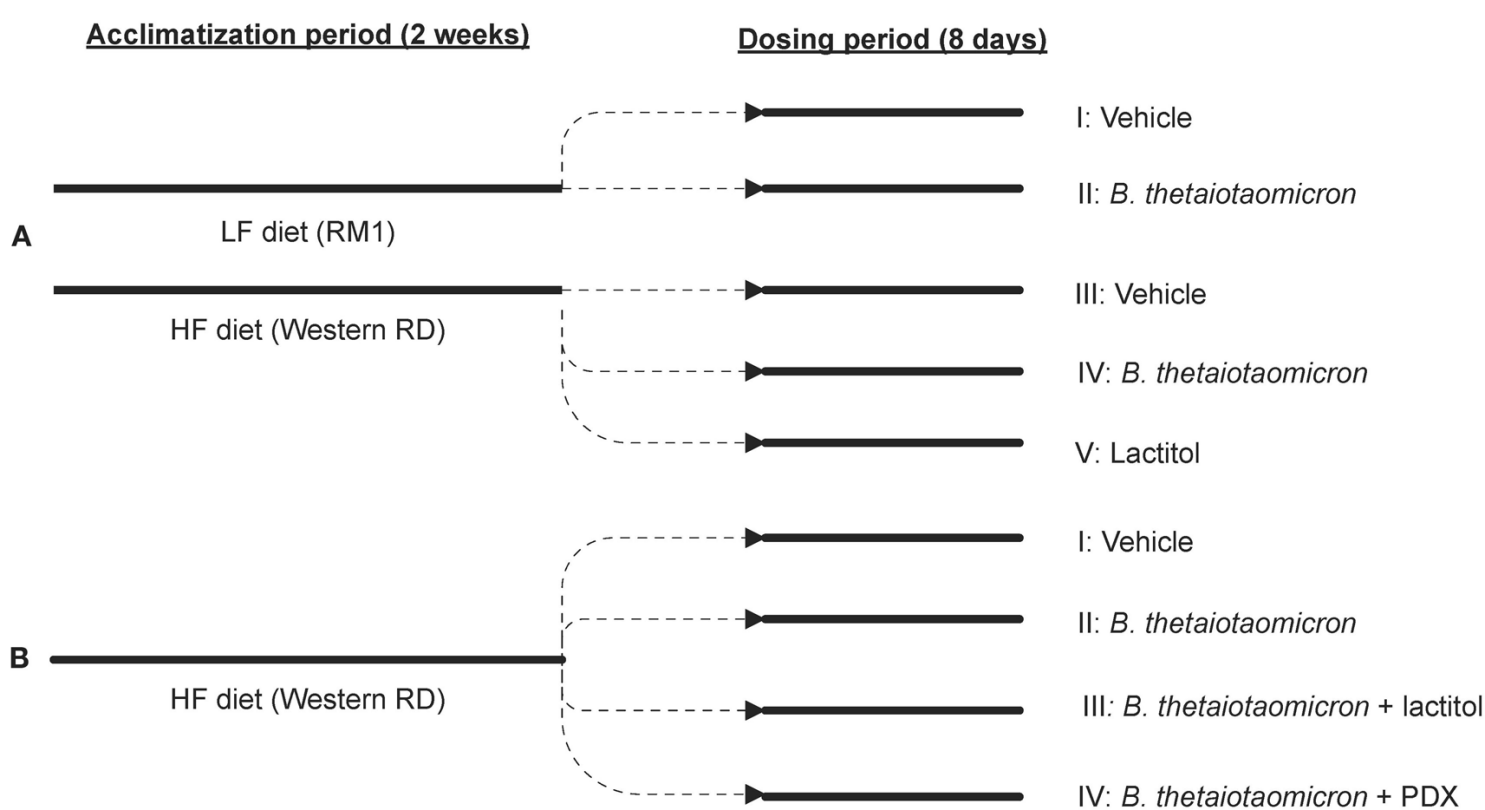
**A flow chart outlining the progression of the animal trials and the arrangement of different treatment groups in Experiments A (A) and B (B)**. 0.9% NaCl functioned as a control vehicle. HF, high-fat diet; LF, low-fat diet; PDX, polydextrose; RD, Rat Diet; RM, rat and mouse maintenance diet.

The animal trial set-up and the arrangement of different treatment groups are presented in Figure 1.

Animals were randomly allocated into five study groups (I–V), 15 animals in each group. Two different diets were used: RM 1 (E) diet (Rat and Mouse Maintenance diet, product code: 801002, Special Diet Services, Witham, UK) was used for the study groups I and II and Western RD (P) diet (Western Rat Diet, product code: 829100, Special Diet Services, Witham, UK) for the study groups III–V. The RM 1 diet contained 7.42% calories from fat and the Atwater fuel energy was 13.75 MJ/kg. The Western RD diet contained 42% calories from fat and the Atwater fuel energy was 19.35 MJ/kg. Therefore, the diets were named LF and HF, respectively (Table 1). Study groups I and III were dosed with a vehicle (0.9% NaCl), groups II and IV with *B. thetaiotaomicron* (10^10^ bacteria/animal/day) in 0.9% NaCl, and group V with lactitol (1.6–1.8 g/animal/day) (Danisco USA Inc., Thomson, IL, USA) in sterile water. *B. thetaiotaomicron* (DSM 2079) was pre-cultured over-night in cooked Meat Medium (Difco, Le Pont de Claix, France) and then cultivated anaerobically over-night in MRS medium (Lab M Limited, Lancashire, UK). The test items used in conjunction with *B. thetaiotaomicron* were prepared in advance and stored at −20°C until the dosage. Lactitol was dissolved in sterile water on the day of the dosage. All test items and the control vehicle were dosed *per os* and administered by oral gavage using Teflon feeding needles (AgnTho’s, Lidingö, Sweden) for 8 days with a volume of 2 ml/rat/day. On study days 1–7, the dosage was carried out in the morning just before feeding period (dark cycle), after which the feed was available *ad libitum* for 5 h. On study day 8, the dosage took place within 2 h before the onset of the dark cycle and the animals were given feed *ad libitum* immediately after the dosage. The feed consumption was measured from each separate cage for 7 days during an acclimatization period and on study days 1–7 (*n* = 5, three animals per cage). Some minor spillage of feed might have occurred through the cage bars and was not recorded. Feed intake was calculated as consumption per 100 g of body weight.

**Table 1:** **The composition of the experimental diets**.

	Experiment A		Experiment B
	LF (RM 1)	HF (Western RD)	HF (Western RD)
Carbohydrates (% cal)	75.09	43	43
Protein (% cal)	17.49	15	15
Fat (% cal)	7.42	42	42
Fiber (%)	4.65	3.5	3.5

*cal, calorie; HF, high-fat diet; LF, low-fat diet; RD, rat diet; RM, rat and mouse maintenance diet*.

#### Experiment B

Animals were allocated into four study groups (I–IV), 25 animals in each group. The HF diet (Western Rat Diet (P), 829100, Special Diet Services, Witham, UK) (Table 1) was used for all study groups throughout Experiment B. Study group I was dosed with a control vehicle (0.9% NaCl) and groups II–IV with *B. thetaiotaomicron* (DSM 2079) (10^10^ bacteria/animal/day) in 0.9% NaCl. In addition to *B. thetaiotaomicron*, groups III and IV received lactitol (2 g/animal/day) (Danisco USA Inc., Thomson, IL, USA) or PDX (2 g/animal/day) (Litesse^®^ Ultra, Danisco USA Inc., Terre Haute, IN, USA), respectively. The test item preparation and the dosage (2 ml/animal/day) were conducted and feed intake (*n* = 10, two to three animals per cage) calculated as described in Experiment A. The feed was available for 5 h after the last dose was given.

### Short-Chain Fatty Acids

Following terminal blood sampling, digesta samples (content of cecum) were taken from all animals, frozen and stored at below −18°C. Gas chromatographic analysis of the SCFAs (acetic acid, propionic acid, butyric acid, isobutyric acid, valeric acid, isovaleric acid, 2-methylbutyric acid) and lactic acid in the rat cecal digesta samples was performed using pivalic acid as an internal standard as described previously (32). SCFAs are expressed as micromole/gram wet weight.

### Dry Matter and Heat Value of Feces

Following terminal blood sampling in Experiment B, fecal samples were collected from the cages (two to three rats per cage), pooled, frozen, and stored at below −18°C. The dry matter of feces was measured gravimetrically after drying the fecal samples in an oven at 105°C for 16 h. The heat value of the dried samples was measured using an adiabatic bomb calorimeter (Parr Instruments, Moline, IL, USA).

### Quantification of *Bacteroides* spp.

Microbial DNA was extracted from the cecum digesta (Experiment A) or fecal samples (Experiment B) by a bead beating step before using the QIAamp DNA stool Mini kit (Qiagen, Hilden, Germany) according to the manufacturer’s instructions. Quantitative PCR (qPCR) was used to quantify the genus *Bacteroides* (including *Bacteroides*–*Provotella*–*Porphyromonas*) using the SYBR green methodology (Core reagent kit, Applied Biosystems, Foster City, CA, USA) in a total volume of 25 μl containing 10 ng of template DNA and 250 nM of the forward primer gBacter_F and reverse primer gBacter_R (33). The amplification and detection of DNA were performed with an ABI 7500 sequencing detection system (Applied Biosystems). To obtain a standard curve, a 10-fold dilution series ranging from 10 pg to 10 ng of DNA from the *B. thetaiotaomicron* (DSM 2079) was added to the qPCR assays. For determination of DNA, triplicate samples were used, and the mean quantity per gram wet weight was calculated.

### Blood Analyses

The studies were designed in a way that enabled several consecutive blood samplings to clarify the kinetics of glucose, triglycerides, insulin, and gut peptides. All blood samples were taken from the lateral tail vein except for the terminal samples that were taken by cardiac puncture under 4% isoflurane anesthesia. On study day, eight blood samples (five animals per group) were taken into the collection tubes (Capiject^®^ or Venosafe™, Terumo^®^ Europe N.V, Leuven, Belgium, EDTA added as an anticoagulant) before the dosage and at 1, 3, 5, and 8 h after the dosage. The only exceptions were the terminal blood samples that were taken to vacuum tubes. The plasma was separated by centrifugation (1600 × *g*, 15 min, RT), and the plasma samples were frozen and stored at −70°C. Insulin and PYY were analyzed from the plasma samples using the MILLIPLEX™ MAP Rat Gut Hormone Panel kit (#RGT-88K, Millipore, Billerica, MA, USA) according to the kit manufacturer’s instructions. The blood glucose was determined from the complete blood samples (five animals per group) using the HemoCue^®^ Glucose 201+ analyzer (HemoCue AB, Ängelholm, Sweden).

In Experiment B, the blood serum samples (10 animals per group) were collected for triglyceride analysis, which was performed with Triglycerides GPO-PAP reagent (Roche Diagnostics GmbH, Mannheim, Germany) and the Roche/Hitachi MODULAR ANALYTICS measuring instrument (Roche Diagnostics GmbH). The serum samples were frozen at −20°C after sampling. Blood triglycerides were not measured in Experiment A.

### Statistical Analysis

The statistical analyses were performed with Prism 5 Version 5.01 (GraphPad Software, Inc., San Diego, CA, USA). One-way ANOVA or two-way ANOVA with Tukey’s multiple comparison test, or unpaired Student’s *t*-test with two-tailed distribution were used, as indicated in the text. For all tests, *p* < 0.05 was considered statistically significant. The areas under the curve (AUCs) of insulin and PYY were calculated using the trapezoidal rule and ignoring the peaks that were <10% of the distance from the minimum or maximum Y values.

## Results

### Body Weight Gain and Feed Intake

The use of a HF diet is a common way to induce obesity in animal models (34). The fat and energy content of the HF diet used in this study (42% calories from fat) were modified so that it resembled the Western human diet.

Lactitol did not have any effect on the daily total fiber supply. However, PDX is widely recognized as soluble dietary fiber (5) and the commercial PDX used in this study (Litesse^®^) is considered to have 90% fiber content. The energy value of PDX is generally recognized as being 1 kcal/g; hence, adding in total 2 kcal/animal/day. The energy value of lactitol is 2 kcal/g, which adds in total 3.2–4 kcal/animal/day.

#### Experiment A

**Figure 2:**
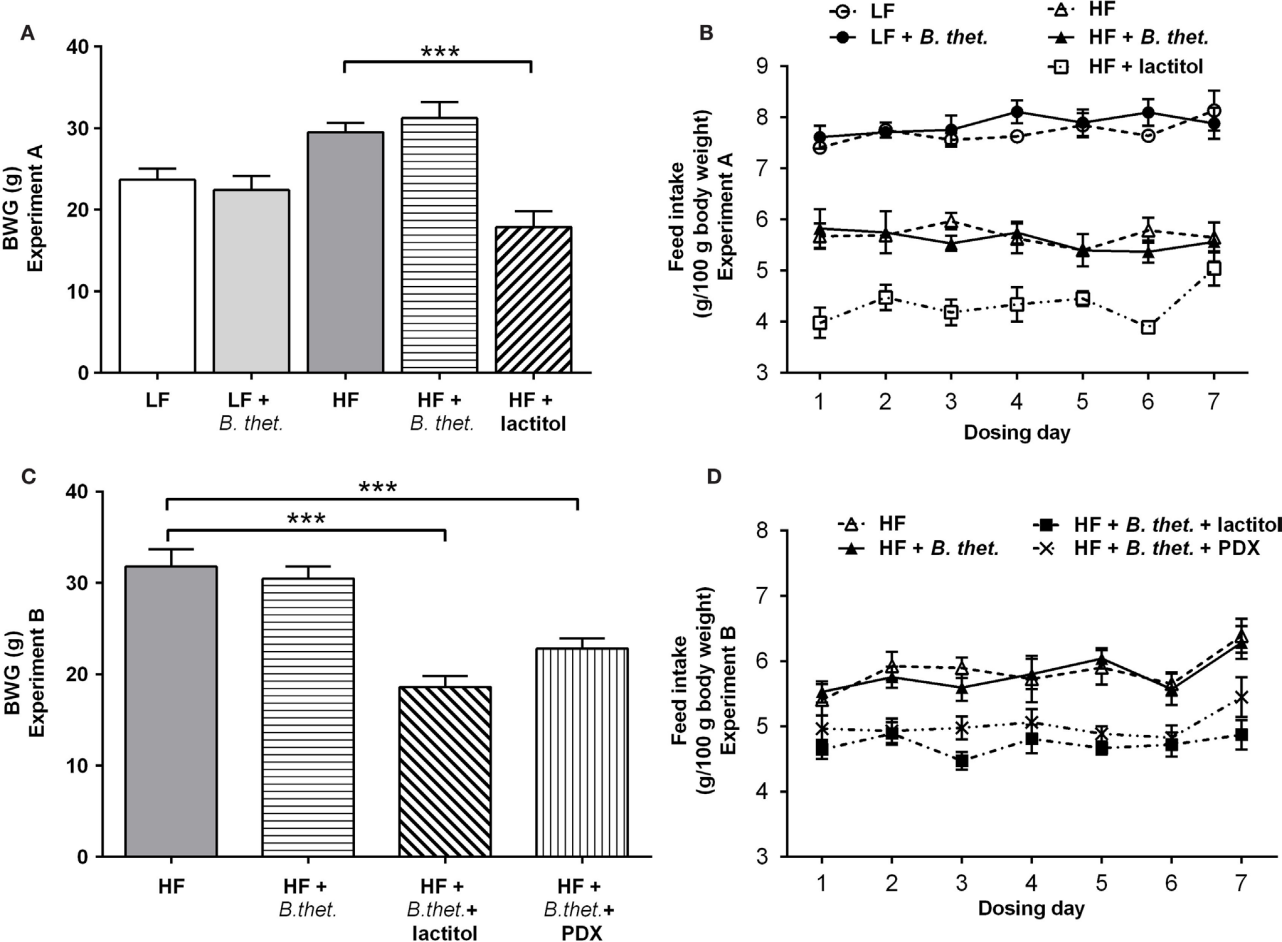
**The body weight gain (BWG) is expressed as grams separately for Experiments A (A) and B (C)**. The actual feed intake values in both experiments are expressed in **(B**,**D)**. The values are means with SEM represented by vertical bars (*n* = 15–25 for BWG, *n* = 5 for feed intake). Significant differences are marked with *asterisks*: ***p < 0.001 (one-way ANOVA). *B. thet., Bacteroides thetaiotaomicron*; HF, high-fat diet; LF, low-fat diet; PDX, polydextrose; SEM, standard error of the mean.

During the acclimatization period, the body weight gain (BWG) increased expectedly in all HF groups when compared to LF groups, and the increases in BWG were statistically significant (*p* < 0.001, one-way ANOVA) when compared to the LF control group (data from acclimatization period not shown). In the lactitol-supplemented HF group, the mean BWG declined by 39% during the 8-day dosing period (*p* < 0.001, one-way ANOVA), when compared to the HF control group (Figure 2A). The LF group supplemented with *B. thetaiotaomicron* did not have any significant effect on the mean BWG during the 8-day dosing period.

Lactitol had the biggest effect on feed intake with nearly a 40% decrease in the mean feed consumption after the acclimatization period (*p* < 0.0001, Student’s *t*-test). In the HF control group and HF group supplemented with *B. thetaiotaomicron*, feed intake decreased by 19 and 18%, respectively (*p* < 0.0001, Student’s *t*-test). The lactitol-supplemented HF group had the lowest mean feed intake when compared to all other groups (*p* < 0.0001, Student’s *t*-test). The two LF groups showed the highest feed intakes among all diet groups; however, there was no statistically significant difference between the LF control group and *B. thetaiotaomicron-*supplemented LF group. The mean feed intake in the HF and LF control groups, on the other hand, differed significantly (*p* < 0.0001, Student’s *t*-test). The actual feed intake values in Experiment A are reported in Figure 2B.

#### Experiment B

In animals fed a HF diet, *B. thetaiotaomicron* supplementation alone did not have any significant effect on the mean BWG. However, when lactitol or PDX were administered together with *B. thetaiotaomicron*, the BWG declined by 41 and 28%, respectively, when compared with the HF control group (*p* < 0.001, one-way ANOVA) (Figure 2C).

The mean feed intake was highest in the HF control and *B. thetaiotaomicron-*supplemented HF groups; however, there was no statistically significant difference between these two groups. The animals administered with the combination of *B. thetaiotaomicron* and lactitol had the lowest mean feed intake (*p* < 0.05, Student’s *t*-test, compared to the other HF groups). The PDX-supplemented HF group consumed on average less feed (*p* < 0.0001, Student’s *t*-test) when compared to the HF control group. The actual feed intake values in Experiment B are reported in Figure 2D.

Some soft feces were noticed in PDX and lactitol-supplemented groups when fecal samples were collected, and one animal per each of these groups had diarrhea during blood sampling.

### SCFAs in Cecum

In Experiment A, the concentrations of total SCFAs, acetic acid, and butyric acid were 35–65% lower in all HF diet-fed animals compared to the LF control animals, but the changes were not statistically significant (one-way ANOVA) (Table 2). In Experiment B, the total SCFA concentrations decreased by 44 and 45% in lactitol and PDX-treated HF groups, respectively (*p* < 0.001, one-way ANOVA), due to a reduction of acetic acid (Table 3). The animals in these groups also received *B. thetaiotaomicron* inoculum, but this did not induce any significant change in the concentration of SCFAs when administered alone. The concentration of branched-chain fatty acids (BCFAs), isobutyric acid, and isovaleric acid decreased in both lactitol and PDX-treated HF groups (*p* < 0.001, one-way ANOVA), and 2-methylbutyric acid in lactitol-treated HF group (*p* < 0.05, one-way ANOVA), when compared to the HF control group. These results are presented in Table 3.

**Table 2:** **Cecal concentrations of SCFAs (*n* = 5) measured 8 h after the last dose in Experiment A**.

Group	LF		LF *B. thet*.		HF		HF *B. thet*.		HF lactitol	
	Mean	SEM	Mean	SEM	Mean	SEM	Mean	SEM	Mean	SEM
**SCFAs (μmol/g)**										
Acetic acid	28.42	11.92	30.66	10.89	17.26	9.42	17.70	9.66	11.83	2.62
Propionic acid	5.19	2.12	6.36	2.52	4.30	2.53	4.73	2.79	6.61	2.81
Butyric acid	21.82	12.35	19.96	11.02	13.07	3.49	13.94	4.33	7.53	2.67
Lactic acid	4.88	3.03	7.46	3.57	3.84	1.88	1.78	0.7	13.68	7.34
**BCFAs (μmol/g)**										
Isobutyric acid	nd		nd		nd		nd		nd	
2-methylbutyric acid	<0.1	0.09	0.14	0.1	0.16	0.07	0.18	0.08	0.13	0.04
Isovaleric acid	0.16	0.11	0.17	0.12	0.17	0.11	0.17	0.11	<0.1	0.03
**Sum of BCFAs (μmol/g)**	0.16	0.2	0.31	0.21	0.33	0.16	0.35	0.18	0.13	0.03
**Sum of all SCFAs (μmol/g)**	60.44	23.65	64.70	20.37	38.73	7.94	38.42	8.36	39.81	7.51

*SCFAs are expressed as μmol/g wet weight. There were no statistically significant differences between different groups in Experiment I. BCFA, branched-chain fatty acid; *B. thet., Bacteroides thetaiotaomicron*; HF, high-fat diet; LF, low-fat diet; nd, not detected; SCFA, short-chain fatty acid; SEM, standard error of the mean*.

**Table 3:** **Cecal concentrations of SCFAs (*n* = 10) and fecal dry matter (*n* = 20) and heat value (*n* = 20) measured 8 h after the last dose in Experiment B**.

Group	HF		HF *B. thet*.		HF *B. thet*. lactitol		HF *B. thet*. PDX	
	Mean	SEM	Mean	SEM	Mean	SEM	Mean	SEM
**Cecal SCFAs (μmol/g)**								
Acetic acid	47.51	2.42	51.28	2.53	21.18***	1.93	22.83***	1.99
Propionic acid	11.95	0.64	12.81	0.8	10.85	1.14	9.39	0.59
Butyric acid	3.63	0.61	4.02	0.88	2.74	0.46	1.92	0.34
Lactic acid	0.31	0.17	0.69	0.2	0.9	0.2	0.43	0.28
**Cecal BCFAs (μmol/g)**								
Isobutyric acid	0.43	0.05	0.52	0.05	<0.1***	0.02	<0.1***	0.02
2-methylbutyric acid	0.16	0.02	0.19	0.03	<0.1*	0.03	0.1	0
Isovaleric acid	0.16	0.03	0.19	0.03	<0.1***	0.01	<0.1***	0.01
**Sum of BCFAs (μmol/g)**	0.75	0.1	0.9	0.1	0.13***	0.03	0.14***	0.03
**Sum of all SCFAs (μmol/g)**	64.5	3.29	69.85	3.69	35.99***	2.93	35.18***	2.5
**Fecal dry matter (%)**	77.05	1.76	76.47	1.9	62.26***	2.12	57.68***	2.08
**Fecal heat value (KJ/g dry matter)**	16.68	0.22	18.38***	0.2	16.00	0.26	17.30	0.19

SCFAs are expressed as μmol/g wet weight. *p < 0.05, **p < 0.01, and ***p < 0.001, compared to the HF control group (ANOVA). BCFA, branched-chain fatty acid; *B. thet., Bacteroides thetaiotaomicron*; HF, high-fat diet; PDX, polydextrose; SCFA, short-chain fatty acid; SEM, standard error of the mean.

### Dry Matter and Heat Value in Feces

Lactitol and PDX in combination with *B. thetaiotaomicron* decreased the dry matter significantly by 19 and 25%, respectively, when compared to the HF control group (*p* < 0.001, one-way ANOVA). The heat value increased by 10% in the *B. thetaiotaomicron*-fed animals, when compared to the HF control group (*p* < 0.001, one-way ANOVA). These data are presented in Table 3.

### *Bacteroides* in Cecal Digesta

**Figure 3:**
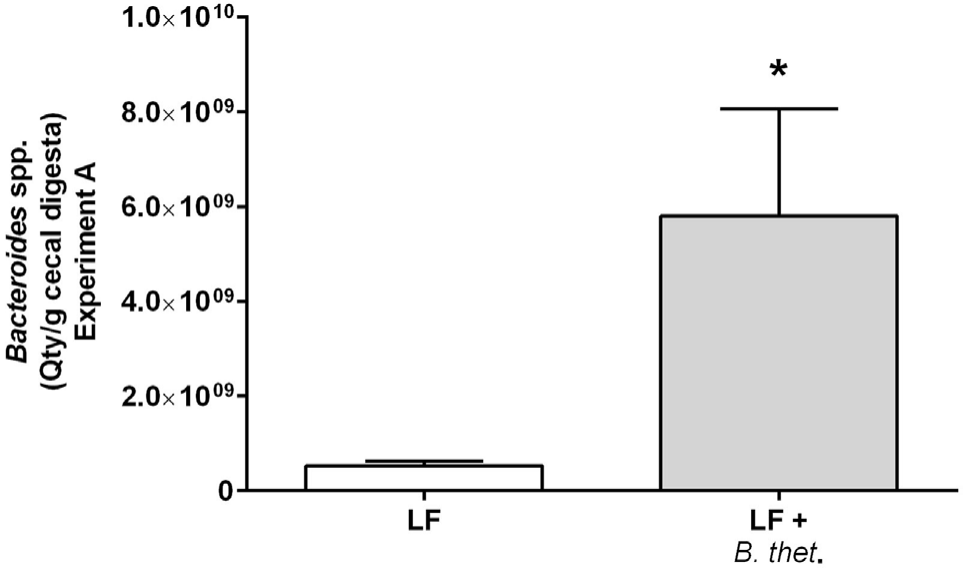
**The prevalence of *Bacteroides* spp. in the cecal digesta of low-fat (LF) fed animals in Experiment A quantified by qPCR**. The values are mean quantity per gram wet weight with SEM represented by vertical bars (*n* = 3). The significant difference is marked with an *asterisk*: *p < 0.05 (one-way ANOVA). *B. thet*., *Bacteroides thetaiotaomicron*; LF, low-fat diet; qPCR, quantitative polymerase chain reaction; SEM, standard error of the mean.

The prevalence of *Bacteroides* spp. in the cecal digesta was quantified by qPCR in only the group of LF diet-fed animals (Figure 3). The high amount of fat in the samples from animals fed the HF diet interfered with the microbial DNA extraction, resulting in unreliable microbial counts. However, the quantity of *Bacteroides* spp. in the cecal digesta in the LF group supplemented with *B. thetaiotaomicron* increased significantly by 11-fold when compared to the LF control group, showing quantities of 9.76 log10 (SEM 9.35 log10) and 8.72 log10 (SEM 7.97 log10) cells per gram cecal digesta (wet weight), respectively (*p* < 0.05, one-way ANOVA).

### Blood Glucose and Triglyceride Response

**Figure 4:**
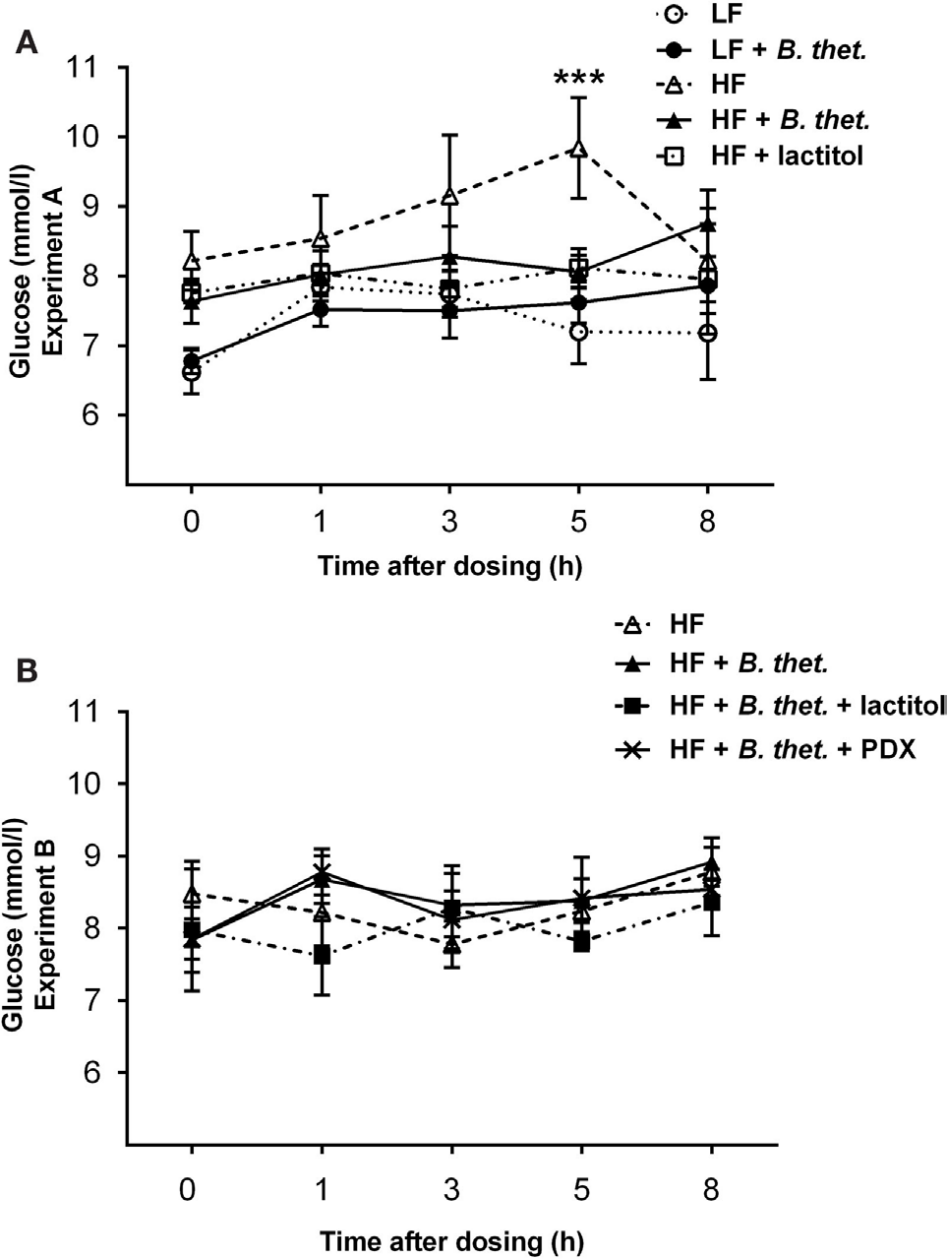
**Postprandial plasma glucose concentrations (millimole/liter) during the last experimental day expressed separately for Experiments A (A) and B (B)**. The values are means with SEM represented by vertical bars (*n* = 4–5). The significant difference is compared to the LF control group (-○-) and marked with *asterisks*: *** *p* < 0.001 (two-way ANOVA). *B. thet*., *Bacteroides thetaiotaomicron*; HF, high-fat diet; LF, low-fat diet; PDX, polydextrose; SEM, standard error of the mean.

Plasma glucose levels increased shortly after the meal. In the LF control group, all glucose values were below 8 mmol/l (Figure 4A), which is within the normal postprandial level in rats (35). Glucose concentrations tended to be higher in the HF control group compared to the LF diet groups, but the only significant deviation from the LF control group was measured at time point 5 h (*p* < 0.001, two-way ANOVA), with the glucose concentration peaking at 9.84 mmol/l (SEM = 0.73) in the HF control group (Figure 4A). Within the LF diet groups, blood glucose levels remained low and there was no difference between the LF control group and the LF group supplemented with *B. thetaiotaomicron*. Blood glucose levels did not differ between the HF diet groups (Figure 4B).

**Figure 5:**
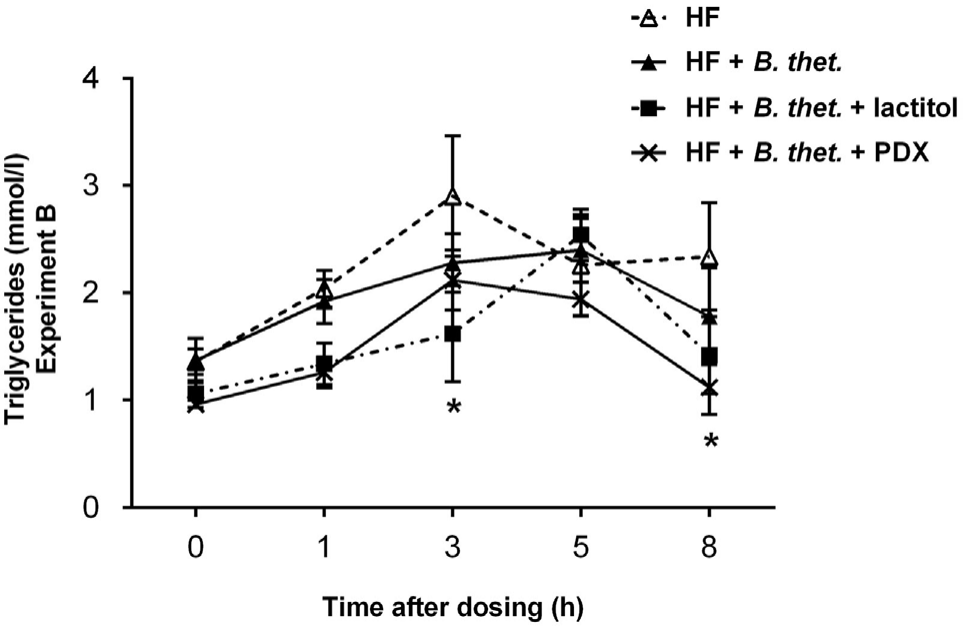
**Postprandial triglyceride concentrations (millimole/liter) in Experiment B**. Samples were taken before the dosage and at 1, 3, 5, and 8 h after the dosage. The values are means with SEM represented by vertical bars (*n* = 5−10). Significant differences are compared to the HF control group (-Δ-) and marked with an *asterisk*: *p < 0.05 (two-way ANOVA). *B. thet., Bacteroides thetaiotaomicron*; HF, high-fat diet; PDX, polydextrose; SEM, standard error of the mean.

Serum triglycerides decreased significantly (*p* < 0.05, two-way ANOVA) in the groups supplemented with the combination of *B. thetaiotaomicron* and lactitol or *B. thetaiotaomicron* and PDX (Figure 5) at time points 3 and 8 h, respectively. In the HF control group, serum triglyceride concentrations peaked earlier (3 h) and were higher than with the *B. thetaiotaomicron* or lactitol-treated groups, which both peaked at 5 h time point. In the PDX-supplemented group, serum triglyceride concentrations peaked also at the 3 h time point. There were no significant differences in the triglyceride concentrations at time point 0.

### Plasma Insulin Response

**Figure 6:**
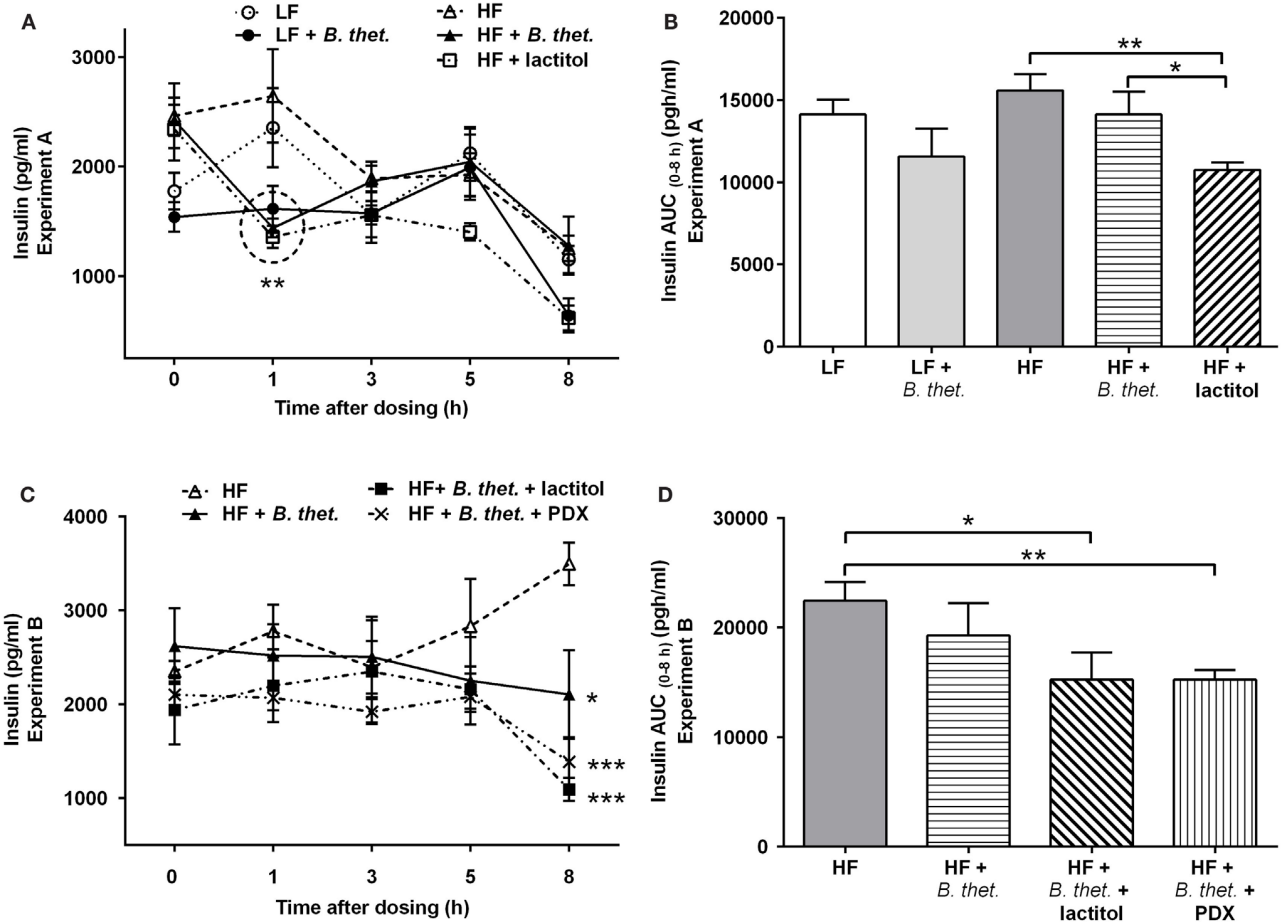
**Postprandial plasma insulin concentrations (picogram/milliliter) during the last experimental day, expressed separately for Experiments A (A) and B (C)**. The areas under the curve (AUC) for insulin concentrations in both experiments are expressed in **(B**,**D)**. The AUC values (0–8 h after the last dose) were calculated using the trapezoidal rule. The values are means with SEM represented by vertical bars (*n* = 4–5). The significant differences are compared to the HF control group (-Δ-) at the same time point or between the indicated groups and marked with *asterisks*: *p < 0.05, **p < 0.01, and ***p < 0.001 (two-way ANOVA for insulin concentrations and Student’s *t*-test for AUC). In figure **(A)**, the statistically significant values are marked with a circle. *B. thet., Bacteroides thetaiotaomicron*; HF, high-fat diet; LF, low-fat diet; PDX, polydextrose; SEM, standard error of the mean.

No significant differences in the fasting insulin levels (time point 0 h) were observed between different groups. Plasma insulin levels increased shortly after the meal initiation in the HF and LF control groups. In the HF groups with *B. thetaiotaomicron* or lactitol supplementation, the acute insulin responses were attenuated, showing decreased insulin levels at time point 1 h, when compared to the fasting values of the same group [*p* = 0.0004 and *p* = 0.012, respectively (Student’s *t*-test)]. Insulin levels in the *B. thetaiotaomicron* and lactitol-supplemented HF groups were lower than in the HF control group at time point 1 h [*p* < 0.01 and *p* < 0.001, respectively (two-way ANOVA)] (Figure 6A). When the insulin AUC (0–8 h) was examined, the HF group administered with lactitol showed significantly lower values than the HF control group or the HF group supplemented with *B. thetaiotaomicron* [*p* = 0.002 and *p* = 0.048, respectively (Student’s *t*-test)] (Figure 6B).

In comparison to the HF control group, the plasma insulin levels decreased 8 h after the meal initiation in animals administered with either *B. thetaiotaomicron* alone, or together with lactitol or PDX [*p* < 0.05, *p* < 0.001, and *p* < 0.001, respectively (two-way ANOVA)] (Figure 6C). No significant differences were seen between the groups at other time points and no significant peaks appeared. The insulin AUC (0–8 h) decreased significantly in lactitol and PDX-enriched groups, compared to the HF control group [*p* = 0.043 and *p* = 0.006 respectively (Student’s *t*-test)] (Figure 6D).

### PYY Release

**Figure 7:**
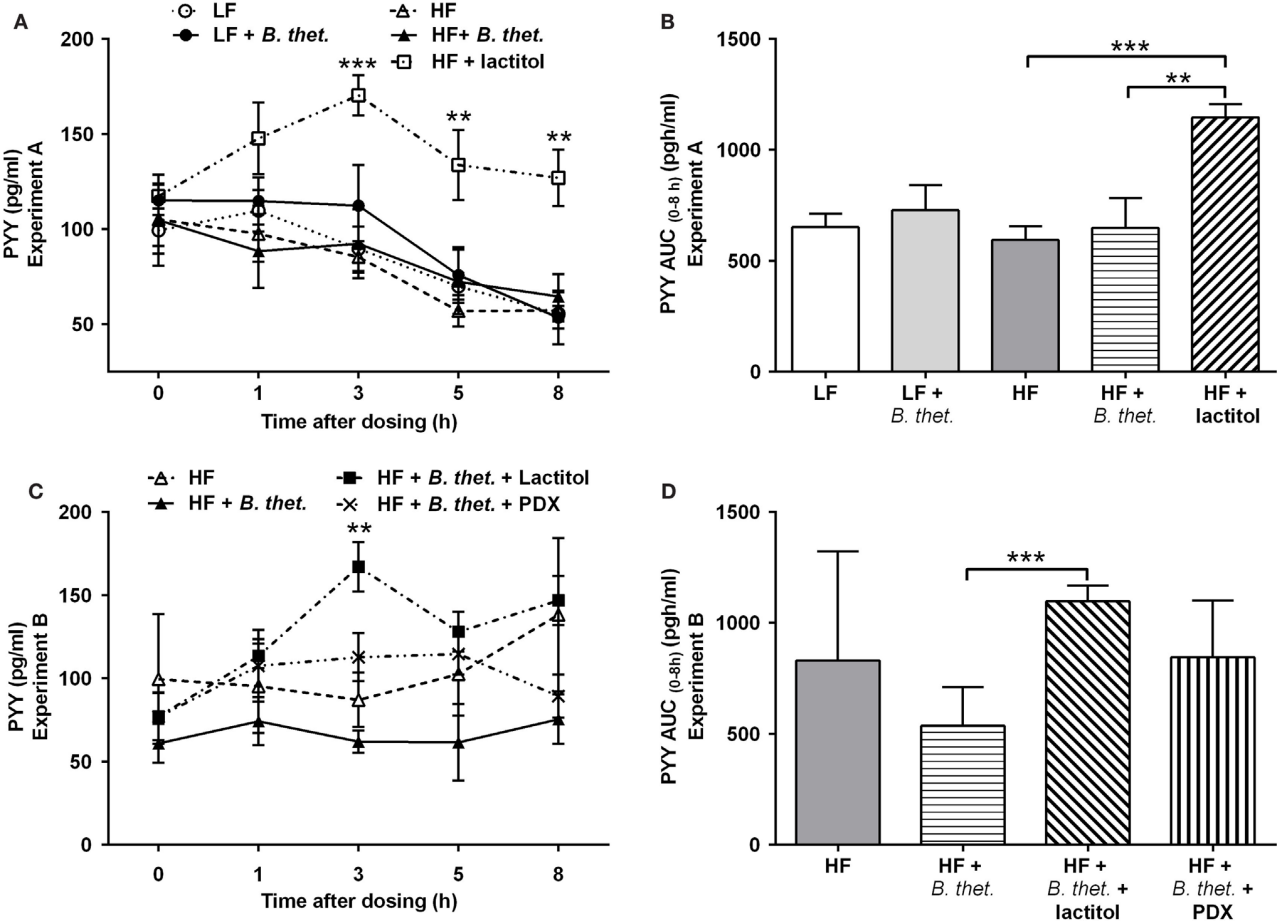
**Postprandial plasma PYY concentrations (picogram/milliliter) during the last experimental day, expressed separately for Experiments A (A) and B (C)**. The areas under the curve (AUC) for PYY concentrations in both experiments are expressed in **(B**,**D)**. The AUC values (0–8 h after the last dose) were calculated using the trapezoidal rule. The values are means with SEM represented by vertical bars (*n* = 4–5). The significant differences are compared to the HF control group (-Δ-) in **(A)**, to *B. thetaiotaomicron* group (-▲-) in **(C)**, or between the indicated groups in **(B**,**D)**, and marked with *asterisks*: **p < 0.01, *** *p* < 0.001 (two-way ANOVA for insulin concentrations and Student’s *t*-test for AUC). *B. thet., Bacteroides thetaiotaomicron*; HF, high-fat diet; LF, low-fat diet; PDX, polydextrose; PYY, peptide tyrosine tyrosine; SEM, standard error of the mean.

Plasma PYY concentrations increased significantly in the lactitol-treated HF group 3–8 h after the final dose, when compared to the HF control group (*p* < 0.01, two-way ANOVA) (Figure 7A). A clear peak in PYY levels was seen 3 h after the application of lactitol. The PYY AUC (0–8 h) in the lactitol-treated group differed significantly from the HF control group and HF group supplemented with *B. thetaiotaomicron* [*p* = 0.0002 and *p* = 0.0098, respectively (Student’s *t*-test)] (Figure 7B). The concentration of PYY increased significantly also in the lactitol group supplemented with *B. thetaiotaomicron* 3 h after the final dose, when compared to *B. thetaiotaomicron* group (*p* < 0.01, two-way ANOVA) (Figure 7C). The PYY AUC (0–8 h) of this combination group also increased significantly, when compared to *B. thetaiotaomicron* group (*p* = 0.0008, Student’s *t*-test) (Figure 7D). *B. thetaiotaomicron* alone did not have any significant effect.

## Discussion

This study investigated whether supplementing a diet with rapidly fermented lactitol and slowly fermented PDX, with or without *B. thetaiotaomicron* inoculum, influences the metabolism and weight maintenance in a rat model. Nutritionally relevant HF diet feeding allows the evaluation of anti-obesity intervention that is fairly similar to the human condition (34). As expected, the mean feed intake differed significantly between LF and HF control groups, and the BWG increased in all HF diet groups during the acclimatization period, when compared to the corresponding LF groups. The HF diet that mimicked the Western human diet was used to induce weight gain and an obese phenotype. The HF diet contained a moderate HF load: 20% (m/m) from milk fat and 0.15% (m/m) of supplementary cholesterol. A similar moderate HF diet has also been used previously in rat studies (36–38). The additive effects of lactitol and PDX on the mean BWG were tested when the rats were fed a HF diet. Both lactitol and PDX supplementations significantly decreased the feed intake and the BWG in HF diet-fed animals. The fiber intake recommendation for humans is 25–35 g/day or 3 g/MJ. The equivalent fiber intake recommendation for an average adult rat would be ~1.5 g/day if the difference in energy consumption between a rat (max 0.49 MJ) and a human (10.47 MJ) is taken into account (27). The doses of lactitol and PDX used in this study (1.6–2 g/animal/day) were close to these recommended values.

The heat value of feces increased in animals supplemented with *B. thetaiotaomicron*, when compared to other groups, indicating that more energy was present in the feces. This would support the initial hypothesis that high amounts of intestinal Bacteroidetes could help to maintain a low body weight. This effect may be mediated through changes in the composition of the gut microbiota or through microbial metabolites. However, the fecal heat value of animals that were given *B. thetaiotaomicron* with lactitol or PDX did not increase. The percentage of fecal dry matter decreased in lactitol and PDX-supplemented animals with more moist feces in these groups.

The potential effect of *Bacteroides* as a probiotic together with fermentable fibers was studied in an animal model. Previously, the synbiotic effects of lactitol with another microbial strain, *Lactobacillus* NCFM^®^, have been successfully tested *in vitro* and in human clinical trials with proven beneficial effects on the gut microbiota and its activity (39, 40). Studies with *B. thetaiotaomicron* have revealed that co-colonization with other microbial species can enhance the efficiency and change the specificity of bacterial polysaccharide fermentation (41). In the present study, the increased fermentation of indigestible carbohydrates was not detected with *Bacteroides* supplementation. In addition, *B. thetaiotaomicron* increased fecal energy value. On the other hand, the BWG and plasma triglyceride concentrations were decreased by lactitol and PDX, when supplemented with *B. thetaiotaomicron*. The dietary fat content is also known to affect the microbiome; i.e., reduced levels of Bacteroidetes are reported in mice fed a HF diet and linked to an increased risk of obesity (23, 42). In our study, the impact of *B. thetaiotaomicron* on the detected higher quantities of *Bacteroides* spp. was significant in LF diet-fed animals, indicating that the dose of *B. thetaiotaomicron* had successfully passed through the gastrointestinal tract. The method of gavaging *B. thetaiotaomicron* has been demonstrated previously as a reliable and efficient way to ensure colonization (41). A limitation of our study, however, was with the use of only one strain of *Bacteroides* spp., while the Bacteroidetes phylum comprises a wide range of strains with different properties. Therefore, the administration of one single strain of *Bacteroides* spp. over a relatively short time proved not to be sufficient enough to counteract the effects of a HF diet.

The HF diet resulted in a marginally lower concentration of total SCFAs compared to a LF containing diet. In animals fed the HF diet, lactitol and PDX decreased the total cecum SCFAs, mostly due to the highly reduced level of acetic acid. Decreased SCFA concentrations with lactitol and PDX have been reported previously in rodents (32, 43). The concentration of BCFAs, including isobutyric and isovaleric acid, was decreased by both lactitol and PDX, indicating less protein fermentation in these groups. A similar decrease in the relative concentration of BCFAs, isobutyric acid and isovaleric acid, by the addition of lactitol and PDX has been noted previously (32). However, no significant effect of the tested supplements on the absolute concentration of butyric acid was seen in the present study. Interestingly, *in vitro* studies with a semi-continuous four-stage colon simulator model have demonstrated that PDX has great potential to increase SCFAs in the gut (7, 44). One explanation to the low SCFA concentration detected in the present study could be the “*snapshot*” nature of the analysis, demonstrating the state of measured SCFAs at a particular time point (8 h after the last dosage) in only a single sample of digesta per animal. Also, an increase of the cecal volume could be one explanation for the reduced SCFA concentrations, as suggested before (32). However, cecal weight was not measured in the present study.

Fermentation of indigestible carbohydrates has been shown to affect glycemic responses and satiety (45). The modulation of the postprandial lipid concentrations by indigestible carbohydrates has not been widely investigated. In the present study, lactitol had already lowered the serum triglycerides 3 h after the last dose was given, when it was used in combination with *B. thetaiotaomicron*. With lactitol, the postprandial triglyceride response was lower and peaked later than with the control group, therefore, weakening the postprandial rise of triglycerides. PDX supplemented with *B. thetaiotaomicron* reduced the serum triglyceride concentration significantly 8 h after the last dose was given. PDX also marginally lowered the immediate triglyceride response. Hence, our data suggest that lactitol and PDX potentially have an attenuating effect on the postprandial triglyceride response in rats fed a HF diet. Previous human studies have also shown that a combination of PDX and lactitol (46), or PDX alone (47) have reduced the level of postprandial serum triglycerides. Since triglycerides are an energy source during metabolism, the reduction of triglycerides in circulation with dietary supplements, such as lactitol or PDX, could be beneficial for weight management and reducing the risk of cardiovascular disease. Even though rat is a commonly used model for metabolic disease studies, conclusions concerning triglyceride responses in humans should be drawn with the great care (48). In addition, differences in postprandial triglyceride responses between genders have been reported in rat studies (49), and this has to be taken into consideration when evaluating the triglyceride effects of potential food ingredients in rats.

Dietary proteins and fibers are known to affect insulin secretion (50, 51). PDX has shown to maintain low postprandial blood glucose levels (26) and lactitol has a low glycemic and insulinemic index (52). In the present study, a HF diet increased glucose levels, when compared to a LF diet that validates the model used in this study. Here, we showed that the pre-meal ingestion of both lactitol and PDX significantly decreased insulin AUC (0–8 h), but had no effect on glucose levels. Interestingly, in both experiments the postprandial plasma PYY levels consistently increased 3 h after the ingestion of lactitol when in combination with *B. thetaiotaomicron*. A similar effect with lactitol has been demonstrated previously (15). However, no direct effect of *Bacteroides* or PDX on PYY levels was noted in the present study. According to previous animal studies, the addition of prebiotic fibers in HF diets fed to rats was able to increase satiety hormone levels and reduce BWG (53). Therefore, managing postprandial glucose and insulin responses with prebiotic supplements could help decrease the risk of metabolic diseases and also play a role in body weight management in humans.

In conclusion, the HF diet had detrimental effects on glucose metabolism and body weight but the two indigestible carbohydrates tested in this study, lactitol and PDX, may provide an additional means of regulating weight management and postprandial metabolism, especially via the triglyceride response. In addition, lactitol significantly increased the PYY satiety hormone levels. However, the oral administration of a single strain of *B. thetaiotaomicron* had a minor or no effect on metabolism.

## Author Contributions

KT planned and supervised the study. K-HH contributed to the design of the study and supervised the PYY analysis. KO analyzed the data and was responsible for writing the manuscript. MS conducted the SCFA, dry matter, and heat value analyses, and contributed to the manuscript. SF supervised the microbial analysis and participated in production of the manuscript. MM was in charge of the animal trials. All authors participated in the interpretation of results, the processing of the final manuscript, and have approved the final version.

## Conflict of Interest Statement

The authors declare that the research was conducted in the absence of any commercial or financial relationships that could be construed as a potential conflict of interest.

## Abbreviations

AUC, area under the curve; BCFA, branched-chain fatty acid; BWG, body weight gain; CCK, cholecystokinin; GLP-1, glucagon-like peptide-1; HF, high fat; LF, low fat; PDX, polydextrose; PYY, peptide tyrosine tyrosine; qPCR, quantitative polymerase chain reaction; SCFA, short-chain fatty acid.
